# Interior of Amylopectin and Nano-Sized Amylopectin Fragments Probed by Viscometry, Dynamic Light Scattering, and Pyrene Excimer Fluorescence

**DOI:** 10.3390/polym12112649

**Published:** 2020-11-11

**Authors:** Lu Li, Jean Duhamel

**Affiliations:** Department of Chemistry, Institute for Polymer Research, Waterloo Institute for Nanotechnology, University of Waterloo, Waterloo, ON N2L 3G1, Canada; l83li@uwaterloo.ca

**Keywords:** amylopectin, pyrene excimer fluorescence, nano-sized amylopectin fragments

## Abstract

Nano-sized amylopectin fragments (NAFs), prepared by extrusion of waxy corn starch, were investigated by viscometry, dynamic light scattering (DLS), and pyrene excimer fluorescence (PEF). NAF57, with a hydrodynamic diameter of 57 nm, was treated with nitric acid to yield three degraded NAFs, which appeared to share the same interior and structural features as amylopectin based on their measured intrinsic viscosity and hydrodynamic diameter. This conclusion was further supported by comparing the efficiency of forming excimer between an excited and a ground-state pyrenyl label covalently attached to the NAFs (Py-NAFs) using their *I*_E_/*I*_M_ ratio of the fluorescence intensity of the excimer (*I*_E_) to that of the monomer (*I*_M_). The overlapping trends obtained for all Py-NAFs and the pyrene-labeled amylopectin samples by plotting the *I*_E_/*I*_M_ ratio as a function of pyrene content provided further evidence that the interior of NAFs and amylopectin shared the same structural features and contained a similar amount of free volume as predicted by the Solution-Cluster (Sol-CL) model. The presence of free volume was validated by adding linear poly(ethylene glycol) (PEG) chains that could not penetrate the interior of Py-NAFs, thus subjecting the Py-NAFs to increased osmotic pressure, which induced their compression and resulted in an increase in *I*_E_/*I*_M_.

## 1. Introduction

Starch nanoparticles (SNPs) can be employed as substitutes of petroleum-based latex used in the paper coating industry, and their potential as food additives or drug carriers is being actively researched [1]. SNPs are expected to retain the main advantages of starch, which is abundant, cost-effective, non-toxic, and renewable [2]. A number of different SNPs have been generated by chemical, biochemical, or mechanical processes. Examples of such processes include acid or enzymatic hydrolysis, high pressure homogenization, ultrasonication, or reactive extrusion [1]. One of the goals of these processes is to reduce the size of starch to increase the dispersibility of the particles in solution and enable the adjustment of their size to meet the requirements for different industry applications [3]. Unfortunately, the properties of starch and SNPs are somewhat limited and they do not meet the vast majority of applications relevant to the polymer industry [4,5,6]. To expand the applicability of SNPs, the chemical modification of SNPs is currently the object of intense research. Most chemical modifications have aimed so far at imparting some hydrophobicity to otherwise hydrophilic SNPs by attaching onto them octenyl succinic anhydride [7,8], oleic and stearic acids [9], polystyrene [10], cholesterol [11], and, more recently, propionic and hexanoic anhydride [12,13]. However, to be most effective, these chemical modifications require the characterization of SNPs not only from a viewpoint pertaining to synthetic polymer particles, based on their hydrodynamic diameter (*D*_h_) and internal density determined from, respectively, dynamic light scattering (DLS) and intrinsic viscosity ([*η*]) measurements, but also, perhaps more importantly, as biopolymer particles, open entities whose interior must be understood at the molecular level in solvents, where they can form a nanodispersion [8]. Such studies are particularly relevant for non-crystalline SNPs such as those produced through extrusion, whereby the crystalline microdomains of the starch granules are fully melted at the high temperatures experienced in the extruder [14]. Upon dispersion in a suitable solvent like dimethyl sulfoxide (DMSO) or water, such SNPs are swollen and their interior, which is exposed to the solvent, can readily react with the chemicals desired for a given chemical modification. A recent study has shown that SNPs produced through extrusion behave differently from typical synthetic polymer particles and that the term particle might be inadequate to describe SNPs [12]. Instead, it was suggested that SNPs could be described as nano-sized starch fragments (NSFs) [12]. The nature of NSFs is still poorly understood and their interior would be worthy of more detailed investigation.

**Figure 1:**
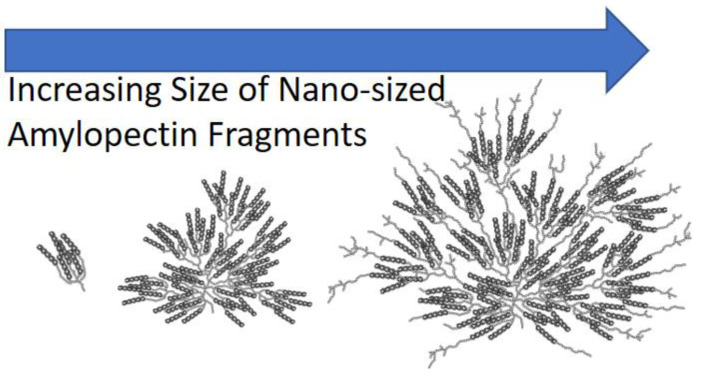
Depiction of the spatial arrangement of the clusters of helices for amylopectin samples of different sizes as predicted by the Solution-Cluster (Sol-CL) model [15].

The Solution-Cluster (Sol-CL) model could help in this regard. It was recently introduced to describe the spatial arrangement of the structural motives of amylopectin dispersed in DMSO [15]. It suggests that amylopectin is constituted of dense clusters of oligosaccharide single helices, which are separated from one another by longer linear oligosaccharides. In this representation, the clusters of oligosaccharide helices represent the building blocks of amylopectin. The clusters of helices are compact, whereas the longer oligosaccharides linking the clusters to one another generate free volume in the amylopectin interior. A larger amylopectin macromolecule should thus have more clusters of helices and more linear oligosaccharide segments, which would generate more free volume. These concepts are illustrated in Figure 1, which shows the spatial arrangement of clusters of helices in amylopectin samples of different sizes.

Based on the Sol-CL model, the smaller nano-sized amylopectin fragments (NAFs) produced from amylopectin should have less excluded volume, making them more rigid and less malleable objects in solution. The study of this effect requires one to measure the size of NAFs and their internal density, as the NAFs are being compressed. To this end, dynamic light scattering (DLS), intrinsic viscosity ([*η*]), and pyrene excimer fluorescence/formation (PEF) were used to determine the hydrodynamic diameter (*D*_h_) of the NAFs, measure their overall density when dispersed in DMSO, and probe the local density experienced by the oligosaccharide side chains constituting the NAFs, respectively. These experiments took advantage of the difference in the length scales probed by each technique. While DLS and viscometry probe macromolecules in their entirety over length scales, which ranged from 8.3 to several hundreds of nanometers for the NAFs and amylopectin samples studied, PEF probes macromolecules over a length scale of 4.6 nm or less, when the dye 1-pyrenebutyric acid (PyBA) is used to label polysaccharides at their C2-hydroxyl [15]. Furthermore, the process of excimer formation, where an excimer is the product of the encounter between an excited and a ground-state pyrene, can be quantified by determining the ratio *I*_E_/*I*_M_ of the fluorescence intensity of the excimer (*I*_E_) over that of the monomer (*I*_M_) [16]. In turn, the *I*_E_/*I*_M_ ratio depends directly on the local concentration of pyrenyl labels ([*Py*]_loc_) covalently attached onto a macromolecule [17]. Changes in the *I*_E_/*I*_M_ ratio of a polysaccharide fluorescently labeled with PyBA indicate changes in [*Py*]_loc_, and thus in the local concentration of the oligosaccharide side chains onto which PyBA is covalently attached.

These concepts were implemented in the present study that provides additional evidence in support of the Sol-CL model introduced earlier [15] and affords a better understanding of the arrangement of oligosaccharide side chains inside NAFs. The experiments described in this paper used the same 21 research-grade NAFs and one amylopectin sample from waxy maize whose characterization has already been reported [15]. In particular, it introduces new experiments, where the degradation of a large NAF by nitric acid was found to yield smaller NAFs, whose *D*_h_ and [*η*] matched the values expected of smaller NAFs. This suggested that the degraded NAFs retained the same arrangement of oligosaccharide side chains in the particle interior as that of the original NAF they were produced from, in the same manner as the NAFs retained the same arrangement of oligosaccharide side chains in their interior as amylopectin from which they were generated. Further support for the common interior shared by NAFs and amylopectin was obtained from PEF measurements which showed that the *I*_E_/*I*_M_ ratios obtained for dispersions in DMSO of Py-Amylopectin, Py-NAF57, Py-NAP20, and Py-NAF8.3, with *D*_h_ values of 225, 57, 20, and 8.3 nm, respectively, clustered around a single master line. This behavior demonstrated that, on the ~4.6 nm length scale for PEF, these four polysaccharides shared a same interior regardless of the overall size of the polysaccharide. The main difference between the polysaccharides was the amount of excluded volume that was expected to increase with increasing polysaccharide size as predicted by the Sol-CL model.

The difference in excluded volume present in the Py-NAFs was further examined by probing the deformability of Py-NAFs in dilute dispersions by increasing the osmotic pressure experienced by the Py-NAFs upon addition of poly(ethylene glycol)s (PEGs) with molecular weights of 0.2, 0.4, 2.0, 5.0, and 10 K to the polysaccharide dispersions in DMSO. On the one hand, the smaller PEGs diffused throughout the Py-NAF interior, and as the solution viscosity increased with increasing PEG concentration, diffusive encounters between pyrenyl labels were reduced and PEF decreased. The larger PEGs, on the other hand, were unable to penetrate the Py-NAF interior, which increased the osmotic pressure experienced by the Py-NAFs as the PEG concentration was increased. Compression of the Py-NAFs raised the local pyrene concentration, which favored PEF. The deformability experienced by the Py-NAFs under increased osmotic pressure was taken as evidence for the existence of excluded volume in the NAF interior. However, the deformability of the Py-NAFs was strongly reduced for smaller NAFs as smaller NAFs exhibited less free volume.

Consequently, this study on Py-NAFs confirmed the existence of oligosaccharide-rich clusters inside NAFs separated by the excluded volume generated by linear oligosaccharide segments bridging the clusters of helices. These considerations are a consequence of the Sol-CL model and are illustrated in Figure 1. This study represents another example of the use of PEF in the characterization of polysaccharides in solution.

## 2. Experimental Section

### 2.1. Instrumentation

The instruments used in this study include an Innova 4000 incubator shaker (NewBrunswik, Edison, NJ, USA) to prepare the polysaccharide dispersions, a Freezone 6 Labconco (Labconco, Kansas City, MO, USA) freeze-dryer for lyophilization, a Photon Technology International LS-100 (PTI, London, ON, Canada) steady-state fluorometer to acquire the fluorescence emission spectra, a time-resolved fluorometer equipped with an IBH 340 nm NanoLED (IBH, Glasgow, Scotland, UK) to acquire the fluorescence decays, a Zetasizer NanoZS (Malvern, Worcester, WR, UK) for dynamic light scattering (DLS) measurements, a Varian Cary 100 Bio spectrophotometer (Varian, Palo Alto, CA, USA) to acquire UV–VIS absorbance spectra, and a Cannon D449-200 Dilution Ubbelohde viscometer (Cannon Instrument Company, State College, PA, USA) for intrinsic viscosity measurements.

### 2.2. Chemicals

Twenty-one research-grade NAFs were supplied by EcoSynthetix (Burlington, ON, Canada) by extruding waxy corn starch, which is >99% amylopectin, under different conditions. Their number average particle hydrodynamic diameter (*D*_h_) ranging from 8.3 to 57 nm was measured by DLS [15]. As a precaution to remove possible additives left over during the production of NAFs, all NAFs were purified by precipitation. Doubly distilled Milli-Q water was obtained from a Millipore Milli-RO 10 Plus or Milli-Q UFPlus (Bedford, MA, USA) system. Dialysis tubing with a 1 kDa molecular weight cutoff (MWCO) was purchased from Spectrum Laboratories Inc. (Spectrum Laboratories Inc., Rancho Dominguez, CA, USA).

### 2.3. Purification of the NAFs by Precipitation

Mixtures of NAFs (5 wt%) in DMSO were placed in a shaker and kept stirring at 250 rpm and 60 °C overnight to generate homogeneous NAF dispersions. After the dispersion cooled to room temperature, the NAFs were precipitated dropwise into ethanol. Suction filtration of the precipitate yielded the NAFs. They were rinsed 4 times with acetone to remove any trace of leftover DMSO. The NAF product was then dried overnight in a vacuum oven at 40 °C.

### 2.4. Dynamic Light Scattering (DLS)

NAFs were dispersed at a concentration of 1 g/L in DMSO by leaving the mixtures overnight in a shaker set at 250 rpm and 60 °C. All DLS measurements were conducted at 25 °C by acquiring the autocorrelation function of the light scattering signal over 5 min in order to obtain a stable baseline. Measurements were repeated 4 times to obtain an average of the number average *D*_h_ determined by DLS for each sample.

### 2.5. Viscometry

Homogenous NAF dispersions with NAF concentration between 1 and 8 g/L in DMSO were obtained by keeping the samples in a shaker set at 250 rpm and 60 °C overnight. A Cannon Ubbelohde viscometer was used to measure the viscosity of the NAF dispersions in DMSO (*η* = 1.99 mPa·s at 25 °C). The temperature of the viscometer was kept at 25 °C during the measurements with a circulating water bath.

### 2.6. Density of PEO(5.0K) Solutions in DMSO

Solutions of PEO(5.0K) in DMSO were prepared. A carefully weighed mass of about 5 g of each PEO(5.0K) solution in DMSO was placed in a cylinder and the volume measured to determine the density of the solution (*d*). The relationship shown in Equation (1) was established between *d* and the weight fraction (*f*_w_) of polymer for PEO(5.0K) concentrations between 0.1 and 41 wt%.

*d* = 1.10 + 0.328 × *f*_w_ (1)

### 2.7. Degradation of NAF57 by Nitric Acid

The largest NAF, namely NAF57 where the number after NAF indicates its *D*_h_, was dispersed in water at a concentration of 10 wt% at 60 °C. The degradation was initiated after adjusting the solution pH to 1 by adding nitric acid. After the desired samples were obtained, the degradation was quenched by adjusting the pH to 6 using a 0.1 M sodium hydroxide aqueous solution. The sodium chloride salt was eliminated by dialysis against double distilled Milli-Q water. The degraded NAFs were recovered by lyophilization.

### 2.8. Synthesis of Pyrene-Labeled NAFs (Py-NAFs)

The preparation of all pyrene-labeled constructs used in this study has been described earlier in detail [15]. 

### 2.9. Deformability of the Polysaccharides

The deformability of Py(4.8)-NAF57 and Py(5.8)-NAF8.3, where the polysaccharide substrate was labeled with 4.8 and 5.8 mol% pyrene and the last number is the *D*_h_ expressed in nanometer, was investigated. The Py-NAFs were dispersed in DMSO using a pyrene concentration of 2.5 × 10^−6^ M equivalent to polysaccharide concentrations of 7.8 and 6.3 mg/L for Py(4.8)-NAF57 and Py(5.8)-NAF8.3, respectively. Py-NAF dispersions with PEG concentrations ranging from 0 to 60 wt% were prepared by adding PEGs with a molecular weight of 0.2, 0.4, 2.0, 5.0, and 10K to the Py-NAF dispersions. The dispersions were heated to 60 °C for 30 min to fully dissolve the PEGs. After the dispersions were equilibrated to room temperature, they were transferred to the fluorescence cell with minimum disturbance to avoid the eventual crystallization of PEGs at high concentration and their steady-state fluorescence spectra were acquired at different PEG concentrations.

### 2.10. Fluorescence Blob Model (FBM) Analysis of the Fluorescence Decays of Py(4.8)-NAF57

The global FBM analysis of the pyrene monomer and excimer fluorescence decays acquired with Py-NAF dispersions in DMSO has been described earlier in detail [15] and are presented in the Supplementary Materials. In brief, the FBM compartmentalizes the Py-NAF macromolecular volume into smaller equivalent subvolumes referred to as *blobs*, which represent the volume probed by an excited pyrenyl label. Random labeling of the NAFs to yield Py-NAF ensured that the pyrenyl labels were randomly distributed among the *blobs* according to a Poisson distribution defined by the average number <*n*> of pyrenyl labels per *blob*. The FBM analysis of the decays retrieved <*n*>, which was then related to the number (*N*_blob_) of anhydroglucose units (AGUs) located inside one *blob* according to Equation (2).

(2) $$N_{\mathrm{blob}}=\frac{1-f_{\mathrm{Mfree}}}{x}\langle n\rangle$$

In Equation (2), *x* represents the molar fraction of pyrene-labeled AGUs in a pyrene-labeled polysaccharide and *f*_Mfree_ is the molar fraction of pyrenyl labels that do not form excimer obtained from the fit of the pyrene monomer fluorescence decay (see the Supplementary Materials).

## 3. Results and Discussion

Several experiments involving DLS, viscometry, and PEF were conducted to probe the interior of NAFs dispersed in DMSO. The results of these experiments provided further support for the notion that NAFs and amylopectin share a similar interior constituted of compact clusters of oligosaccharide helices held together by long linear oligosaccharide segments, which generate excluded volume in the macromolecular volume.

### 3.1. Viscometry and DLS Experiments

**Figure 2:**
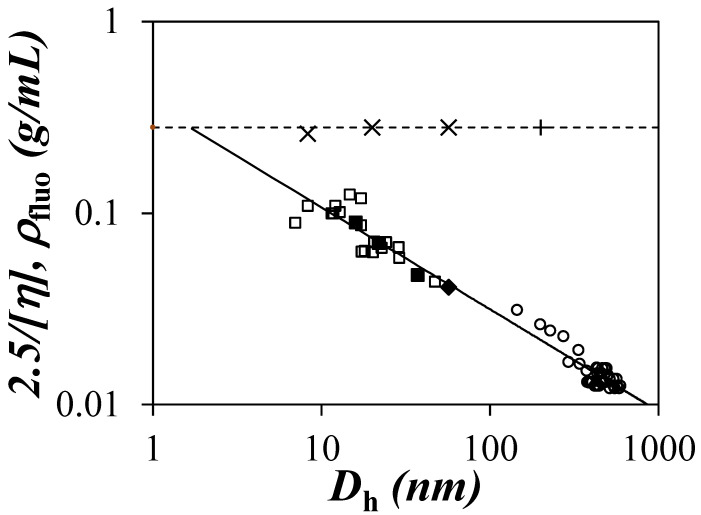
Plot of 2.5/[*η*] obtained by viscometry and *ρ*_luo_ obtained by pyrene excimer fluorescence (PEF) as a function of *D*_h_. Data for 2.5/[*η*]: (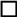) the NAFs, (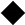) NAF57, (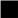) the degraded NAF57 samples, and (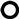) a series of samples derived from amylopectin in dimethyl sulfoxide (DMSO). Data for *ρ*_fluo_: (×) NAFs and (+) amylopectin [12]. [*η*] = 6.9 × *D*_h_^0.53^.

One of the implications of the Sol-CL model is that degradation of amylopectin should generate nano-sized amylopectin fragments (NAFs) with an interior that should reflect that of amylopectin, as suggested in Figure 1. This was indeed observed earlier for a series of 21 research-grade NAFs with different *D*_h_ values that were prepared by extruding waxy corn starch, which is >99% amylopectin, under different conditions [15]. As described earlier, iodine binding experiments and ^1^H NMR measurements demonstrated that the NAFs were amylose free and had the same chemical composition as the amylopectin, from which they were prepared [15]. When [*η*] was plotted against *D*_h_ for the NAFs, as seen in Figure 2, all data points were found to cluster around a master curve [15] that lined up with those obtained for different samples prepared from amylopectin as described in an earlier publication [18]. Taking 2.5/[*η*] as the density of the polysaccharide particles since it equals *M*/(*V_h_* × *N_A_*), a substantial decrease in the density of the polysaccharide was observed with increasing *D*_h_ of the polysaccharides, as seen in Figure 2. The fact that all [*η*] and *D*_h_ values clustered around a same master line in Figure 2 was taken as evidence that the NAFs shared a similar internal architecture, resulting in an internal density given by 2.5/[*η*] that would decrease with increasing *D*_h_. The decrease in polysaccharide density with increasing polysaccharide size was viewed as a clear indication that the interior of amylopectin and the NAFs prepared from it had some excluded volume whose contribution increased with increasing polysaccharide size.

PEF experiments also determined that the density (*ρ*_fluo_) experienced by the pyrenyl labels remained constant with the polysaccharide size, as seen in Figure 2, and was one to two orders of magnitude higher than the quantity 2.5/[*η*] [15]. This discrepancy was attributed to the difference in length scales probed by fluorescence and viscometry, with fluorescence probing solely the denser regions corresponding to the clusters of helices in the interior of the polysaccharides, whereas 2.5/[*η*] represented the density of the entire polysaccharides.

Interestingly, as seen in Figure 2, the master line obtained for the NAFs generated through the breakdown of amylopectin induced by the extrusion of waxy corn starch could be reproduced by taking the largest sample among the 21 NAFs that had a *D*_h_ of 57 nm (NAF57) and subjecting it to a nitric acid treatment. Application of the nitric acid treatment to NAF57 over different reaction times yielded three degraded NAFs. As shown in Figure 2, the degraded NAF57 products also clustered along the same master curve as the other NAFs. This result was taken as evidence that the variation in particle density with size shown in Figure 2 was due to the highly branched nature of amylopectin, from which all these NAFs were created and which was reproduced in all the NAFs but on a smaller length scale, as suggested in Figure 1. Interestingly, it also suggested that nitric acid targeted the loose linear segments connecting the clusters of helices for degradation, whereas the more compact clusters of helices might have provided some protection against chemical degradation by making the polysaccharide backbone less accessible to nitric acid. As a result, the nitric acid treatment generated smaller NAFs that retained the same internal structure as their parent amylopectin molecule. A better experimental design would have used amylopectin as the starting material for degradation. However, the amylopectin sample used in the study had a broad size distribution, making its characterization by DLS difficult and, thus, the determination of its *D*_h_ challenging. Instead, the [*η*] value of 122 mL/g determined experimentally for the amylopectin sample in DMSO was used with the master curve in Figure 1 to determine that it had a *D*_h_ of 225 nm. Its degradation with nitric acid generated highly polydisperse products, which further complicated the analysis of the DLS results.

### 3.2. Steady-State Fluorescence of the Pyrene-Labeled NAFs and Amylopectin

**Figure 3:**
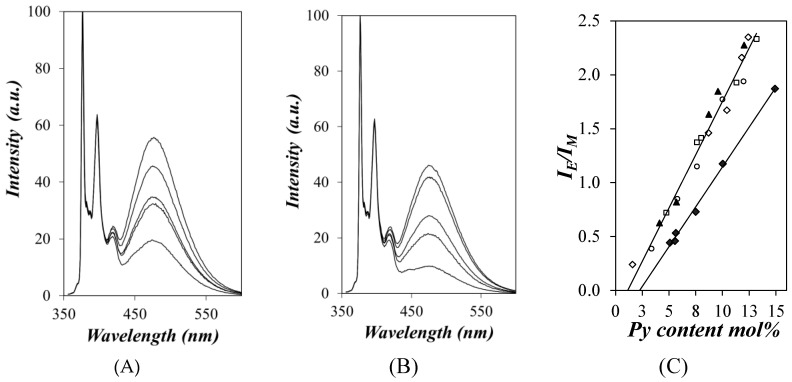
Fluorescence spectra of (**A**) Py-NAF57 and (**B**) Py-NAF8.3 in DMSO. From bottom to top, the pyrene content equals (**A**) 4.8, 7.6, 8.0, 11.3, and 13.2 mol% and (**B**) 3.4, 5.8, 7.6, 10.0, and 12.0 mol%. (**C**) Plot of *I*_E_/*I*_M_ as a function of pyrene content for (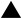) Py-Amylopectin [15], (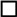) Py-NAF57, (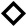) Py-NAF20, (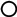) Py-NAF8.3, and (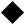) Py-Amylose [19].

Amylopectin, NAF57, NAF20, and NAF8.3 were labeled with different amounts of 1-pyrenebutyric acid to yield Py-Amylopectin, Py-NAF57, Py-NAF20, and Py-NAF8.3, respectively. The steady-state fluorescence spectra were acquired for all samples in DMSO, and those of the Py-NAF57 and Py-NAF8.3 samples are shown in Figure 3A and Figure 3B, respectively. The fluorescence intensity was normalized at 376 nm, which corresponds to the 0–0 transition of the pyrenyl label and is set to an arbitrary value of 100. The fluorescence spectra were acquired for samples with a pyrene concentration of 2.5 × 10^−6^ M that corresponded to polysaccharide concentrations lower than 26 mg/L, dilute enough to avoid aggregation that would otherwise lead to intermolecular excimer formation. The fluorescence spectra show the sharp peaks of the pyrene monomer between 370 nm and 410 nm as well as the broad emission of the pyrene excimer centered at 480 nm. More pyrene excimer was formed with increasing pyrene content due to the increased probability of encounters between the pyrenyl labels covalently attached to the polysaccharide backbone. The fluorescence spectra were further analyzed by integrating the fluorescence signal corresponding to the pyrene monomer (*I*_M_) and excimer (*I*_E_) to yield the *I*_E_/*I*_M_ ratio, which is plotted as a function of pyrene content in Figure 3C.

When studying pyrene-labeled polymers, the *I*_E_/*I*_M_ ratio is typically viewed as a measure of the efficiency of pyrene excimer formation. All *I*_E_/*I*_M_ ratios obtained for the Py-Amylopectin, Py-NAF57, Py-NAF20, and Py-NAF8.3 samples clustered along a master line when plotted as a function of pyrene content, indicating similar PEF efficiencies for these four samples. This master line reflected stronger PEF efficiency with increasing pyrene content as would be expected, since a higher pyrene content promotes more pyrene–pyrene encounters, and thus more PEF. The master line also indicated that the four branched polysaccharides generated more excimer compared to the Py-Amylose samples that yielded lower *I*_E_/*I*_M_ ratios [19]. 

The stronger PEF efficiency observed for the Py-Amylopectin and Py-NAF samples was attributed to the highly branched nature of amylopectin, as depicted in Figure 1, which brought the pyrenyl labels closer to each other, thus enhancing PEF. What was remarkable in the trend shown in Figure 3C for Py-Amylopectin, Py-NAF57, Py-NAF20, and Py-NAF8.3 was that all *I*_E_/*I*_M_ ratios landed on the same master curve regardless of the size of the polysaccharide considered, which ranged from the *D*_h_ of 225 nm for the amylopectin sample down to the more than 20-fold smaller *D*_h_ of 8.3 nm for NAF8.3. This observation implied that the environment probed by an excited pyrene was the same inside these four polysaccharides. Since the reach of a 1-pyrenebutyryl label attached on the C2-hydroxyl of an AGU has been found to equal 2.3 nm [15], it implied that each pyrenyl label probed a same volume ~4.6 nm in diameter, and that the local concentration of pyrenyl labels in this volume remained the same regardless of whether the polysaccharide construct was amylopectin or an NAF prepared from amylopectin.

The behavior observed in Figure 3C for the *I*_E_/*I*_M_ ratios is in perfect agreement with the Sol-CL model depicted in Figure 1. Because PEF is both short ranged (it occurs over ~4.6 nm) and highly efficient for the four branched polysaccharides, as seen in Figure 3C, PEF must be arising from the denser regions of the polysaccharides, which would be the clusters of helices. That PEF was unaffected by the overall size of the polysaccharide indicates that the same clusters of helices must be present in all these branched polysaccharides regardless of their actual macromolecular size. The PEF experiments, which probe a macromolecule over a ~4.6 nm length scale for a PyBA derivative, nicely complemented the combination of DLS and [*η*] measurements, which probed the macromolecule over length scales that ranged from 8.3 to 225 nm for the samples studied. Finally, the fact that the *I*_E_/*I*_M_ ratios obtained for the Py-Amylopectin [15], Py-NAF57, Py-NAF20, and Py-NAF8.3 samples were the same for a same pyrene content but remained consistently larger than the *I*_E_/*I*_M_ ratios obtained for Py-Amylose [19] suggests that PEF may respond to the architecture of a specific polysaccharide and could be used to differentiate between the origins of two unknown polysaccharides.

### 3.3. Osmotic Pressure Experiments

The data presented so far indicated that amylopectin and the NAFs derived from it exhibited excluded volume generated by the linear oligosaccharide segments bridging the clusters of helices, whose number decreased with decreasing *D*_h_. This feature suggested that amylopectin could be compressed if long linear polymer chains were added to the amylopectin dispersion, whose large size would prevent them from entering the amylopectin interior, thus increasing the osmotic pressure experienced by the amylopectin interior. Since the *I*_E_/*I*_M_ ratio is proportional to the local pyrene concentration [17], compression of Py-Amylopectin by increased osmotic pressure generated in the polysaccharide dispersion upon addition of long linear polymers should result in an increased *I*_E_/*I*_M_ ratio. This was indeed observed when large PEG(10K) and PEG(20K) samples were added to a dispersion of amylopectin labeled with 4.1 mol% of PyBA (Py(4.1)-Amylopectin) in DMSO [15]. The large PEG samples could not penetrate the interior of Py(4.1)-Amylopectin and a substantial increase in *I*_E_/*I*_M_ was observed upon increasing the polymer concentration.

**Figure 4:**
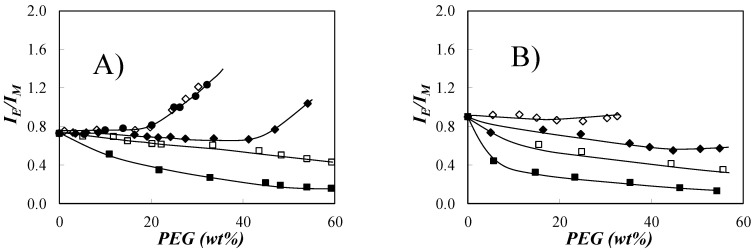
Plots of *I*_E_/*I*_M_ as a function of poly(ethylene glycol) (PEG) concentration for (**A**) Py(4.8)-NAF57 and (**B**) Py(5.8)-NAF8.3. PEGs with *M*_n_ of (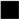) 0.2, (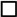) 0.4, (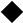) 2.0, (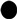) 5.0, and (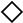) 10 K. [Py(4.8)-NAF57] = 9.1 mg/L, [Py(5.8)-NAF8.3] = 7.7 mg/L.

While these experiments confirmed the deformability of amylopectin, they also suggested that smaller NAFs prepared from amylopectin should be harder to deform since they would have less excluded volume. To assess whether this would indeed be the case, five PEGs, namely PEG(0.2K), PEG(0.4K), PEG(2.0K), PEG(5.0K), and PEG(10K), with molecular weights of 0.2, 0.4, 2.0, 5.0, and 10 K, respectively, were added to a dilute dispersion of Py(5.8)-NAF8.3 and Py(4.8)-NAF57 labeled with 5.8 and 4.8 mol% pyrene, respectively. The *I*_E_/*I*_M_ ratio of the dispersions of the pyrene-labeled polysaccharides was monitored as a function of PEG concentration, and the results are shown in Figure 4.

The trends obtained by plotting *I*_E_/*I*_M_ as a function of PEG concentration in Figure 4A,B are best explained by evoking Equation (3), which relates *I*_E_/*I*_M_ with the bimolecular rate constant (*k*_diff_) for diffusion-controlled PEF and the local pyrene concentration ([*Py*]_loc_) experienced by an excited pyrene bound to the macromolecule. Since *k*_diff_ reflects a diffusion-controlled process, *k*_diff_ is inversely proportional to the solution viscosity, which increases dramatically with increasing PEG concentration [15]. Furthermore, the increase in viscosity with PEG concentration is more pronounced for the longer PEGs. The decrease in *k*_diff_ with increasing PEG concentration resulted in the decrease in the *I*_E_/*I*_M_ ratio with increasing PEG(0.2K) concentration for Py(4.8)-NAF57 and Py(5.8)-NAF8.3 in Figure 4A and Figure 4B, respectively.

(3) $$\frac{I_{E}}{I_{M}}\propto k_{\mathrm{diff}}\times {\left[Py\right]}_{\mathrm{loc}}$$

Surprisingly for a process that was diffusion controlled, the *I*_E_/*I*_M_ ratio decreased less with increasing concentration of larger PEGs despite the fact, that they yielded a larger solution viscosity [15]. Since *k*_diff_ decreases with increasing PEG size at a given PEG concentration because it is inversely proportional to solution viscosity, the only explanation for the larger *I*_E_/*I*_M_ ratios observed with increasing PEG size was that [*Py*]_loc_ in Equation (3) must have increased to offset the decrease in *k*_diff_. The increase in [*Py*]_loc_ with increasing PEG size suggested that the larger PEGs could not penetrate the crowded interior of the NAFs, thus applying osmotic pressure to the polysaccharides. Compression of the NAFs concentrated the pyrene labels inside the NAF interior, which yielded a larger *I*_E_/*I*_M_ ratio. This behavior is similar to that observed for the deswelling of Py-Amylopectin with large PEG(10K) [15] or that of a swollen polymeric network that can be induced by free polymers excluded from the network [20,21,22,23,24,25]. 

Indeed, the effect that the osmotic pressure generated by excluded PEG chains has on poly(*N*-isopropylacrylamide) (PNIPAM) microgels and macrogels has been investigated [20,21,22,23,24]. For a given PEG concentration, the deswelling ratio first increased with increasing molecular weight of the PEGs, before becoming independent from PEG MW when the free chains were fully excluded from the gel particles [24]. The results were actually in good agreement with the standard expression of the osmotic pressure derived by Flory and Huggins, whereby the osmotic pressure generated by the free chains depends on their volume fraction rather than their molecular weight [26]. It must be emphasized that this theory assumed that penetration of free chains into the particles could not occur. When the free chains in solution are small enough that they can diffuse into the gel particles, the chemical potential difference between the interior and exterior of the particles is partially balanced, leading to a reduction in the deswelling ratio.

A similar effect was observed with Py-NAF57 for which the *I*_E_/*I*_M_ ratios obtained for Py-NAF57 with PEG(5.0K) and PEG(10K) overlapped and showed a more pronounced increase with increasing PEG concentration than that obtained with PEG(2.0K). The difference in behavior between PEG(5.0K) and PEG(2.0K) indicated that the mesh size of NAF57 corresponding to the distance separating the clusters of oligosaccharide helices was between the size of PEG(2.0K) and PEG(5.0K), since PEG(5.0K) was fully excluded from NAF57, whereas PEG(2.0K) partially penetrated it. Using the end-to-end distance *r*_EE_ (r_EE_ = 0.119 × $\sqrt{M_{n}}$ × 0.707 nm) [27] for PEG chains in water, the *r*_EE_ values of 3.8 and 5.9 nm for PEG(2.0K) and PEG(5.0K), respectively, suggested that the mesh size of NAF57 lies between these two values at 5.0 (± 1.0) nm. As it turns out, this NAF mesh size agrees remarkably well with a prediction made earlier that the oligosaccharide single helices inside the clusters are separated by an interhelical distance of 2.8 nm and that the clusters must be separated by at least 3.2 nm [15]. The smaller PEG(0.2K) could easily fit within the helices inside and outside a cluster so that the addition of PEG(0.2K) to the NAF dispersions in DMSO increased the solvent viscosity throughout the NAF interior, leading to the decrease in *I*_E_/*I*_M_. In contrast, the larger PEG(5.0K) and PEG(10K) were excluded from the interhelical and intercluster voids so that they remained outside the NAFs, where they increased the osmotic pressure resulting in larger *I*_E_/*I*_M_ ratios due to an increase in [*Py*]_loc_.

Moreover, it is noteworthy that the compression induced by the osmotic pressure generated by the larger PEG chains would only occur for NAFs possessing sufficient excluded volume. As shown in Figure 4A, the larger Py-NAF57 sample had enough excluded volume to undergo a volume reduction in the presence of PEG(10K), whereas Py-NAF8.3 did not. In fact, for an interhelix distance (*d*_h-h_) of ~2.8 nm predicted by fluorescence, a cluster of seven helices arranged in a hexagonal array, as shown in Figure 1, would have a diameter of ~6 nm and a thickness of 9 nm, equal to the thickness of crystalline lamellae found in dry amylopectin [28]. Consequently, Py-NAF8.3 with a *D*_h_ of 8.3 nm had a dimension comparable to that of a single cluster of helices and, as such, its compression was more limited compared to that of Py-NAF57 that had much more excluded volume as was observed experimentally in Figure 4B.

### 3.4. Time-Resolved Fluorescence of Py(4.8)-NAF57

**Figure 5:**
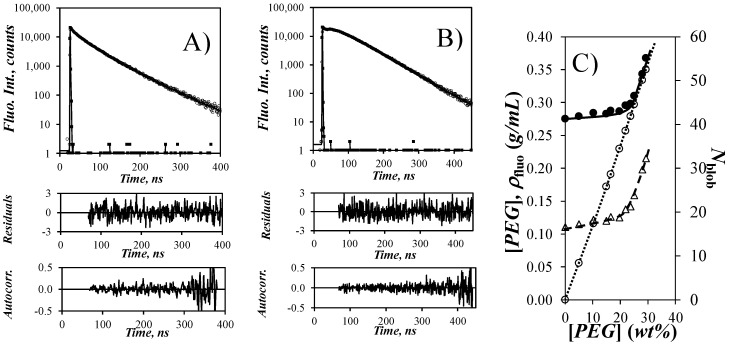
Fluorescence decays of the pyrene (**A**) monomer and (**B**) excimer of Py(4.8)-NAF57 in DMSO with 0.32 g/mL PEG(5.0K). (**C**) Plot of (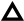, right axis) *N*_blob_, (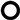) PEG(5.0K) concentration, and (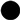) *ρ*_fluo_ as a function of PEG(5.0K) wt%. [Py(4.8)-NAF57] = 9.1 mg/L.

The strong increase in *I*_E_/*I*_M_ observed as a function of increasing PEG(5.0K) and PEG(10K) concentration for Py(4.8)-NAF57 in Figure 4A reflected an increase in [*Py*]_loc_ induced by an increase in osmotic pressure sensed by NAF57, which brought the oligosaccharide side chains bearing pyrenyl labels closer to each other. Fluorescence decays were acquired for the pyrene monomer and excimer of a 9.1 mg/L Py(4.8)-NAF57 dispersion in DMSO to which an increasing amount of PEG(5.0K) was added. The fluorescence decays were fitted according to the FBM. The fits were excellent as shown in Figure 5A,B. The parameters retrieved from the FBM analysis of the decays are listed in Tables S1–S3 in the Supplementary Materials. The FBM analysis yielded <*n*>, which was used to calculate *N*_blob_ according to Equation (2). *N*_blob_ was plotted as a function of the PEG(5.0K) weight fraction in Figure 5C. *N*_blob_ increased slightly from 17 to 19 when the PEG(5.0K) concentration increased from 0 to 20 wt% before increasing much more steeply from 19 to 32 when the PEG(5.0K) concentration increased from 20 to 29.3 wt%. Interestingly, the steep increase in *N*_blob_ for PEG(5.0K) concentrations greater than 20 wt% matched the steep increase in *I*_E_/*I*_M_ in Figure 4A, confirming that the increase in [*Py*]_loc_ probed by *I*_E_/*I*_M_ brought the oligosaccharide side chains closer to each other, resulting in the increase in *N*_blob_. The shorter interhelical distance (*d*_h-h_) separating the oligosaccharide side chains enabled more dye encounters between the pyrenyl labels, which was reflected by the increase in *N*_blob_.

As it turns out, an earlier publication [15] established Equation (4) to relate *N*_blob_ to *d*_h-h_ for oligosaccharide single helices closely packed in a hexagonal array. In turn, *d*_h-h_ can be employed to determine the density (*ρ*_fluo_) probed by the pyrenyl labels using Equation (5). *ρ*_luo_ was plotted as a function of PEG(5.0K) concentration in Figure 5C. *ρ*_fluo_ remained more or less constant and equal to 0.28 g/mL until the PEG(5.0K) concentration approached 25 wt%, at which point *ρ*_fluo_ increased with increasing PEG(5.0K) concentration.

The concentration of PEG(5.0K) in these DMSO solutions was determined by multiplying the density of the PEO(5.0K) solutions in DMSO given by Equation (1) with the weight fraction of the polymer (*f*_w_). Remarkably, the increase in *ρ*_fluo_ coincided with the increase in PEG(5.0K) concentration, resulting in a perfect match between [PEG] and *ρ*_fluo_ for PEG(5.0K) concentrations above 25 wt%. The increase in *ρ*_fluo_ from its original value of 0.28 g/mL when the PEG(5.0K) concentration approached this *ρ*_fluo_ value is also indicative of an increase in osmotic pressure, with the local density inside the particles increasing to match the larger density generated outside the particles. This result further supports the conclusion drawn earlier that the increase in *I*_E_/*I*_M_ in Figure 4A and *N*_blob_ in Figure 5C resulted from an increase in osmotic pressure. Finally, the trend shown in Figure 5C provides further validation for the PEF-based methodology implemented earlier to determine *ρ*_fluo_ [15].

(4) $$N_{\mathrm{blob}}=10.39+\frac{27.04}{1+{\left(d_{h-h}/28.06\right)}^{17.34}}$$

(5) $$\rho_{\mathrm{fluo}}\left(g/\mathrm{mL}\right)=\frac{2.5\times 10^{-12}}{d_{h-h}^{2}\left(innm^{2}\right)}$$

## 4. Conclusions

The experiments described in this paper have consistently indicated that NAFs derived from the chemical or mechanical degradation of amylopectin have an interior that is similar to that of amylopectin as suggested by the Sol-CL model. The Sol-CL model suggests that the interior of NAFs and amylopectin is constituted of compact clusters of oligosaccharide helices linked to each other by linear oligosaccharides, which generate excluded volume. This mixture of dense and compact domains rationalizes many of the experimental observations made with amylopectin in solution and should provide a better understanding of the changes in the properties experienced by amylopectin after being subjected to different chemical modifications. Of particular interest was the inaccessibility of the interior of amylopectin and the NAFs derived from it to PEG chains having a *D*_h_ larger than ~5.0 nm. The deformability experiments presented in Figure 4 suggest that such large macromolecules are excluded from the interior of amylopectin and should only interact with its surface. These conclusions were further supported by using time-resolved fluorescence measurements to measure the density of the NAF57 sample as it was compressed by increasing concentrations of PEG (5.0K). The Sol-CL model appears well suited to rationalize the results described in this paper for the behavior of different polysaccharides derived from amylopectin.

## Supplementary Materials

The following are available online at https://www.mdpi.com/2073-4360/12/11/2649/s1, Equations S1–S14: Equations for fluorescence decay analysis; Table S1: Parameters retrieved from the global FBM analysis with Equations (S1) and (S2) of the monomer decays of Py(5.8)-NAF57 in aerated DMSO with PEG5K., Table S2: Parameters retrieved from the global FBM analysis with Equations (S1) and (S2) of the excimer decays of Py(5.8)-NAF57 in aerated DMSO with PEG5K. *τ*_ES_ is fixed to equal 3.5 ns in the analysis, Table S3: Fractions of all pyrene species of Py(5.8)-NAF57 in aerated DMSO with PEG5K calculated from *f*_Mdiff_, *f*_Mfree_, fEdiff, *f*_Ek2_, and *f*_EE0_.
