# Supplementary material for: Interior of Amylopectin and Nano-Sized Amylopectin Fragments Probed by Viscometry, Dynamic Light Scattering, and Pyrene Excimer Fluorescence
Source: Polymers (Basel). 2020 Nov 11;12(11):2649. doi: 10.3390/polym12112649 (PMC7696867; doi:10.3390/polym12112649)
Supplement: Supplementary file 1 [file polymers-12-02649-s001.pdf]

polymers

MDPI

Article

Supplementary Materials for

Interior of Amylopectin and Nano-Sized Amylopectin Fragments Probed by Viscometry, Dynamic Light Scattering, and Pyrene Excimer Fluorescence

Lu Li and Jean Duhamel *

Department of Chemistry, Institute for Polymer Research, Waterloo Institute for Nanotechnology, University of Waterloo, Waterloo, ON N2L 3G1, Canada; l83li@uwaterloo.ca
* Correspondence: jduhamel@uwaterloo.ca

Received: 23 October 2020; Accepted: 8 November 2020; Published: date

A. Global analysis of the monomer and excimer fluorescence decays according to the fluorescence blob model and equations for the molar fractions of the different pyrene species

The fluorescence decays of the monomer and excimer of the Py-NAF57 samples in DMSO with PEG5K were fitted globally with Equations S1 and S2. Figure 5A,B in the main text shows that the fits were excellent with residuals and autocorrelation of residuals randomly distributed around zero. The χ^2 values listed in Tables S1–S3 were all smaller than 1.3, also indicating good fit quality.

$$[Py*]_{(t)} = [Py^*_{diff}]_{(t)} + [Py^*_{k2}]_{(t)} + [Py^*_{free}]_{(t)} = [Py^*_{diff}]_o \, exp\left(-\left(A_2 + \frac{1}{\tau_M}\right)t - A_3(1 - exp(-A_4 t))\right) + \left([Py^*_{k2}]_o + [Py^*_{diff}]_o e^{-A_3} \sum_{i=0}^{\infty} \frac{A_3^i}{i!} \frac{A_2 + iA_4}{A_2 + iA_4 - k_2}\right) exp\left(-\left(k_2 + \frac{1}{\tau_M}\right)t\right) - [Py^*_{diff}]_o e^{-A_3} \sum_{i=0}^{\infty} \frac{A_3^i}{i!} \frac{A_2 + iA_4}{A_2 + iA_4 - k_2} exp\left(-\left(A_2 + iA_4 + \frac{1}{\tau_M}\right)t\right) + [Py^*_{free}]_o \, exp\left(-\frac{t}{\tau_M}\right)] \quad (S1)$$

$$[E*]_{(t)} = [E0*]_{(t)} + [ES*]_{(t)} = k_2\left(\left([Py^*_{k2}]_o + [Py^*_{diff}]_o e^{-A_3} \sum_{i=0}^{\infty} \frac{A_3^i}{i!} \frac{A_2 + iA_4}{A_2 + iA_4 - k_2}\right) \times \frac{exp\left(-\frac{t}{\tau_{E0}}\right) - exp\left(-\left(k_2 + \frac{1}{\tau_M}\right)t\right)}{k_2 + \frac{1}{\tau_M} - \frac{1}{\tau_{E0}}} + [Py^*_{diff}]_o e^{-A_3} \sum_{i=0}^{\infty} \frac{A_3^i}{i!} \frac{A_2 + iA_4}{A_2 + iA_4 - k_2} \frac{exp\left(-\left(A_2 + iA_4 + \frac{1}{\tau_M}\right)t\right.}{A_2 + iA_4 + \frac{1}{\tau_M}} \cdot [E0*]_o \times exp\left(-\frac{t}{\tau_{E0}}\right) + [ES*]_o \times exp\left(-\frac{t}{\tau_{ES}}\right) \quad (S2)$$

Equations (S1) and (S2) are the same equations that were used in earlier studies of pyrene-labeled polysaccharides.[1,2]. The parameters A_2, A_3, and A_4 used in Equations S1 and S2 are functions of the parameters <n>, k_{blob}, and k_e×[*blob*], which are, respectively, the average number of ground-state pyrene labels per *blob*, the rate constant describing the diffusive motion of two structural units bearing an excited pyrenyl label and a ground-state pyrenyl label located inside a same *blob*, and the product of the rate constant k_e of ground-state pyrenes exchanging between *blobs* and the local concentration of *blobs* inside the polysaccharide macromolecular volume. The expressions of A_2, A_3, and A_4 are given

in Equations (S3)a–c. The encounter between one structural unit bearing an excited pyrenyl label and another bearing a ground-state pyrenyl label led to the rapid rearrangement of the pyrenyl labels, which formed an excimer with a large rate constant k_2.

$$A_2 = < n > \times \frac{k_{blob} k_e [blob]}{k_{blob} + k_e [blob]} \tag{S3a}$$

$$A_3 = < n > \times \left(\frac{k_{blob}}{k_{blob} + k_e [blob]} \right)^2 \tag{S3b}$$

$$A_4 = k_{blob} + k_e \times [blob] \tag{S3c}$$

The global fluorescence blob model analysis (GFBMA) was carried out first for all the decays of Py(4.8)-NAF57 in DMSO with PEG5K with a floating rate constant k_2. The rate constant k_2 obtained for each Py(4.8)-NAF57 sample was averaged, and the GFBMA of the decays was repeated with the value of k_2 being fixed to its average of 2.5×10^8 s^{-1}. This procedure, which has been in place since 2010 [3], has been found to substantially decrease the spread in the parameters retrieved from the GFBMA. These parameters are listed in Tables S1–S3.

The pre-exponential factors in Equations (S1) and (S2) provided information about the relative molar fractions of the different pyrene species present in the solution. Five pyrene species are expected to be present in a Py(4.8)-NAF57 dispersion. These pyrene species are the pyrene labels Py_{free}^*, which do not form excimer and emit as if they were free in solution with the natural lifetime of the pyrene monomer (τ_M), Py_{diff}^*, which are attached on a structural unit that diffuses in solution with a rate constant k_{blob} until it encounters another structural unit bearing a ground-state pyrene label where it turns into the species Py_{k2}^*, which forms excimer upon rapid rearrangement of the pyrene labels with a rate constant k_2, and $E0^*$, which are the pyrene labels that emit as excimer produced either by the rapid rearrangement of two nearby pyrenyls with a rate constant k_2 or by the direct excitation of a pyrene aggregate. Finally, the species ES^* is a short-lived species that only appeared in the excimer decays and is often observed when pyrene excimer formation takes place in restricted geometries as found for pyrene covalently attached onto the rigid polysaccharide backbone. The pre-exponential factors in Equation S1 provided information about the molar fractions f_{Mdiff}, f_{Mk2}, and f_{Mfree} of the pyrene species that contributed to the monomer decays and whose expressions are given in Equations (S4)–(S6).

$$f_{Mdiff} = \frac{[Py_{diff}^*]_o}{[Py_{diff}^*]_o + [Py_{k2}^*]_o + [Py_{free}^*]_o} \tag{S4}$$

$$f_{Mk2} = \frac{[Py_{k2}^*]_o}{[Py_{diff}^*]_o + [Py_{k2}^*]_o + [Py_{free}^*]_o} \tag{S5}$$

$$f_{Mfree} = \frac{[Py_{free}^*]_o}{[Py_{diff}^*]_o + [Py_{k2}^*]_o + [Py_{free}^*]_o} \tag{S6}$$

Similarly, the pre-exponential factors in Equation S2 yielded the molar fractions f_{Ediff}, f_{Ek2}, f_{EE0}, and f_{EES} of the pyrene species represented in the excimer decays. The expression of these fractions are provided in Equations (S7)–(S10).

$$f_{Ediff} = \frac{[Py_{diff}^*]_o}{[Py_{diff}^*]_o + [Py_{k2}^*]_o + [E0*]_o + [ES*]_o} \tag{S7}$$

$$f_{Ek2} = \frac{[Py_{k2}^*]_o}{[Py_{diff}^*]_o + [Py_{k2}^*]_o + [E0\,*]_o + [ES\,*]_o} \tag{S8}$$

$$f_{EE0} = \frac{[E0*]_o}{[Py^*_{diff}]_o + [Py^*_{k2}]_o + [E0*]_o + [ES*]_o} \quad \text{(S9)}$$

$$f_{EES} = \frac{[ES*]_o}{[Py^*_{diff}]_o + [Py^*_{k2}]_o + [E0*]_o + [ES*]_o} \quad \text{(S10)}$$

The short-lived species ES* disappeared quasi immediately after excitation from the long-lived excimer decay and its contribution was not included to determine the molar fractions f_{diff}, f_{k2}, f_{free}, and f_{E0} of the pyrene species in solution as is typically done with a GFBMA. The expression of these fractions is given in Equations S11 – S14.

$$f_{diff} = \frac{1}{1 + (f_{Mk2}/f_{Mdiff}) + (f_{Mfree}/f_{Mdiff}) + (f_{EE0}/f_{Ediff})} \quad \text{(S11)}$$

$$f_{k2} = f_{diff}\frac{f_{Mk2}}{f_{Mdiff}} \quad \text{(S12)}$$

$$f_{free} = f_{diff}\frac{f_{Mfree}}{f_{Mdiff}} \quad \text{(S13)}$$

$$f_{E0} = f_{diff}\frac{f_{EE0}}{f_{Ediff}} \quad \text{(S14)}$$

The values of all the parameters retrieved from the global decay analysis of the pyrene monomer and excimer with Equations S1 and S2 are listed in Tables S1–S3.

B. List of parameters retrieved from the fluorescence decay analysis

Table S1. Parameters retrieved from the global FBM analysis with Equations S1 and S2 of the monomer decays of Py(5.8)-NAF57 in aerated DMSO with PEG5K.

Sample	[PEG]	f_{Mdiff}	f_{Mfree}	k_{blob} (× 10^7 s ^{1})	<n>	f_{k2}	$k_{\mathrm{e}}[blob]$ (× 10^6 s ^{1})	$\chi 2$
Py(5.8)-NAF57<br>$_{\mathrm{M}}$ = 92 ns<br>k_2 = 2.5 × 10^8 s ^{1}	0.29	0.75	0.04	1.08	1.59	0.21	8.3	1.05
	0.27	0.76	0.04	1.05	1.47	0.19	6.5	1.12
	0.26	0.78	0.03	1.19	1.17	0.18	7.3	1.03
	0.24	0.73	0.06	1.35	1.08	0.22	8.2	1.02
	0.22	0.79	0.01	1.26	1.01	0.20	6.2	1.07
	0.20	0.77	0.01	1.40	0.95	0.18	8.2	0.99
	0.17	0.81	0.01	1.34	0.92	0.18	7.2	1.15
	0.15	0.81	0.03	1.46	0.89	0.16	14.6	1.16
	0.10	0.76	0.03	1.35	0.90	0.21	13.5	1.06
	0.05	0.80	0.02	1.39	0.85	0.17	23.9	1.06
	0.00	0.80	0.04	1.62	0.82	0.16	10.4	1.09

Table S2. Parameters retrieved from the global FBM analysis with Equations S1 and S2 of the excimer decays of Py(5.8)-NAF57 in aerated DMSO with PEG5K. τ_{ES} is fixed to equal 3.5 ns in the analysis.

Sample	[PEG]	τ_{E} (ns)	f_{Ediff}	f_{Ek2}	f_{EE0}	f_{ES}	$\chi 2$
Py(5.8)-NAF57<br>$_{\mathrm{M}}$ = 92 ns<br>k_2 = 2.5 × 10^8 s ^{1}	0.29	50.8	0.50	0.14	0.09	0.28	1.05
	0.27	50.9	0.53	0.14	0.09	0.24	1.12
	0.26	52.1	0.56	0.13	0.07	0.24	1.03
	0.24	51.4	0.48	0.14	0.00	0.38	1.02
	0.22	51.8	0.55	0.14	0.03	0.28	1.07
	0.20	50.8	0.57	0.13	0.05	0.25	0.99

	0.17	50.6	0.56	0.12	0.03	0.29	1.15
	0.15	50.2	0.59	0.12	0.06	0.22	1.16
	0.10	48.6	0.57	0.16	0.01	0.27	1.06
	0.05	48.7	0.60	0.13	0.05	0.22	1.06
	0.00	49.0	0.59	0.12	0.06	0.23	1.09

Table S3. Fractions of all pyrene species of Py(5.8)-NAF57 in aerated DMSO with PEG5K calculated from f_{Mdiff}, f_{Mfree}, f_{Ediff}, f_{Ek2}, and f_{EE0}.

Sample	[PEG]	f_{diff}	f_{free}	f_{E0}	f_{k2}
Py(5.8)-NAF57<br>τ_M = 92 ns<br>$k_2 = 2.5 \times 10^8$ s^{-1}	0.29	0.66	0.04	0.11	0.19
	0.27	0.67	0.04	0.12	0.17
	0.26	0.71	0.03	0.09	0.17
	0.24	0.73	0.06	0.00	0.22
	0.22	0.76	0.01	0.04	0.19
	0.20	0.75	0.01	0.06	0.17
	0.17	0.77	0.01	0.04	0.17
	0.15	0.74	0.03	0.08	0.15
	0.10	0.75	0.03	0.01	0.21
	0.05	0.75	0.02	0.06	0.16
	0.00	0.74	0.04	0.08	0.15

References

1. Li, L.; Duhamel, J. Conformation of Pyrene-Labeled Amylose in DMSO Characterized with the Fluorescence Blob Model. *Macromolecules* **2016**, *49*, 7965-7974.
2. Li, L.; Kim, D.; Zhai, X.; Duhamel, J. A Pyrene Excimer Fluorescence (PEF) Study of the Interior of Amylopectinin Dilute Solution. *Macromolecules* **2020**, *53*, 6850–6860.
3. Ingratta, M.; Mathew, M.; Duhamel, J. How Switching the Substituent of a Pyrene Derivative from a Methyl to a Butyl Affects the Fluorescence Response of Polystyrene Randomly Labeled with Pyrene. *Can. J. Chem.* **2010**, *88*, 217-227.

© 2020 by the authors. Licensee MDPI, Basel, Switzerland. This article is an open access article distributed under the terms and conditions of the Creative Commons Attribution (CC BY) license (http://creativecommons.org/licenses/by/4.0/).
